# Results of a Patient Reported Experience Measure (PREM) to measure the rare disease patients and caregivers experience: a Spanish cross-sectional study

**DOI:** 10.1186/s13023-021-01700-z

**Published:** 2021-02-05

**Authors:** Mercedes Guilabert, Alba Martínez-García, Marina Sala-González, Olga Solas, José Joaquín Mira

**Affiliations:** 1 Health Psychology Department, Calitè Research Group, Miguel Hernández University, Elche, Spain; 2 Calitè Research Group, Miguel Hernández University, Elche, Spain; 3 Health Policy Management, Toledo, Spain; 4 Alicante-Sant Joan Health District, Alicante, Spain; 5 Red de Investigación en Servicios de Salud en Enfermedades Crónicas, REDISSEC, Alicante, Spain

**Keywords:** Rare diseases, Patient experience, Integrated care, Quality assurance, Questionnaire

## Abstract

**Objective:**

To measure the experience of the person having a rare disease in order to identify objectives for optimal care in the health care received by these patients. Methods. A cross-sectional study was conducted in Spain involving patients associated with the Spanish Rare Diseases Federation [Federación Española de Enfermedades Raras] (FEDER). A modified version of the PREM IEXPAC [Instrumento para evaluar la Experiencia del Paciente Crónico] instrument was used (IEXPAC-rare-diseases). Scores ranged between 0 (worst experience) and 10 (best experience).

**Results:**

A total of 261 caregivers (in the case of paediatric population) and patients with rare diseases (response rate 54.4%) replied. 232 (88.9%) were adult patients and 29 (11.1%) caregivers of minor patients. Most males, 227 (87%), with an average age of 38 (SD 13.6) years. The mean time since confirmation of diagnosis was 7.8 (SD 8.0) years. The score in this PREM was 3.5 points out to 10 (95%CI 3.2–3.8, SD 2.0). Caregivers of paediatric patients scored higher, except for coordination of social and healthcare services.

**Conclusions:**

There are wide and important areas for improvement in the care of patients with rare diseases. This study involves a first assesment of the experience of patients with rare diseases in Spain.

## Background

Diseases with a lower prevalence of 0.65% are known as rare [1]. Most of the approximately 7000 of rare diseases which are known have a genetic cause and are diagnosed in childhood [2, 3]. The paradox of rare diseases lies in the fact that, although each of the pathologies classified as rare affects a very small number of people, the total number of people having a rare disease is high [4, 5].

People having a rare disease share a long journey through the set of assistance centres until diagnosis [6–9]. Rare diseases test the capacity of health systems as these people (particularly in the case of childhood) need different responses to meet their physical, social, and psychological needs in a cocherent, integrated, and effective way [4, 10, 11].

Health organizations that pursue to a person-centered care systematically measure the experiences of their patients in the course of the care they receive. However, the problem of people having a rare disease has been little studied so far [12, 13] and less in Spain. The limited research results have applied methods based on surveys or qualitative techniques. These results are similar and highlight the delays in diagnosis, the demand for information, and the burden of the family compared to other chronic processes. This last one is due to the uncertainty of low frequency, that relatives normally have more information about the disease than most professionals, that there is no adequate coordination between care levels as this role is exercised by relatives and that the care received is fragmented [1, 14, 15].

Patient-Reported-Experience-Measure (PREM) is a methodological approach little applied in the case of patients with rare diseases, despite its extensive application in patients with chronic diseases [16]. This is a measure of a patient's perception of their personal experience of the healthcare they have received [17, 18]. The PREMs are instruments that have been designed with the opinion and perspective of patients. Moreover, the PREMs have demonstrated its positive associations with health outcomes [4, 19].

As far as we know, in our country no studies have been carried out using standardised instruments (PREM instruments) to evaluate the experience of patients having a rare disease or their caregivers, analysing the response capacity and the degree of integration of healthcare provision at different levels of care. The aim of this study was to measure the experience of the person having a rare disease in order to identify objectives for optimal care in the health care received by these patients.

## Method

A cross-sectional study was carried out between June and December 2018. This study was approved by Ethics Committee of Clinical research at the San Juan of Alicante University General Hospital (18/303 on 5 February 2018).

### Subjects

A total of 480 subjects were invited to participate considering an error sampling of 5% and lost expected responses of 20%.

### Procedure

The Spanish Rare Diseases Federation [Federación Española de Enfermedades Raras] (FEDER) collaborated in this study sending a set of his affiliates invitations to reply. They received a mail explaining the aim and the procedure for replying. This message included a link directing to the online questionnaire to be self-administered by subjects. Voluntary response and anonymized treatment were emphasized. A remainder was done to promote the response.

### Instrument

A modified version of the IEXPAC instrument [20] was applied. This instrument includes 11 items, plus an additional item for patients recently hospitalized. IEXPAC explores characteristics and content of interactions between patients and professionals designed to improve outcomes, the ability of individuals to cope with their diseases, manage their own care and improve their well-being, based on interventions mediated by healthcare professionals, and experience of use the new forms of patient interaction with the healthcare system. For each item, patients responded on a 5-point Likert scale (range from never to always).

Four questions of this instrument were modified to adjust to the rare diseases context (reinforcing the measure of patient-centered-care, support for engagement, and integrate care) and four specific items were added to explore the experience with care in case of emergency care, homecare, being receiving support from social services and information received about diagnosis. Adaptation was done by a selection of specialist working on rare diseases and the research team applying consensus technique. Responses were transformed in scores following criteria applied by Orozco et al. [21] using the IEXPAC considering always (score 10), mostly (7.5), sometimes (5), seldom (2.5) or never (0). The overall score was calculated as the sum of individual scores for the 11 common items divided by 11 between 0 (worst experience) and 10 (best experience). This procedure allowed to compare data with the national study conducted by Orozco et al. including 1618 chronic patients having diabetes mellitus, human immunodeficiency virus infection, inflammatory bowel disease, or rheumatic diseases. Additionally, percentages of subjects who answered with the always option was calculated. These percentages allows identification of range in which improvement is needed and compare data with the Spanish studies conducted asking chronic patients[9] and caregivers of chronic patients (in that studies relatives have a diagnosis of Alzheimer, mental illness, and Chronic Obstructive Pulmonary Disease) [22] using the same scale.

This version of the IEXPAC was assessed by a set of patients representatives and their wording changes suggested were introduced to assure content validity and legibility. Additionally, metric properties of this instrument was assessed to assure the validity (applying exploratory factorial analysis) and reliability (using Cronbach’s Alpha and Rho coefficient) of the measurement conducted in this study. The reliability measures using Cronbach’s Alpha of this version of the IEXPAC instrument for rare diseases was 0.87 and coefficient Rho was 0.86 (for the 11 elements applied in all cases). Items converged in two factors explaining 59.4 of total variance (“Appendix 1”).

### Statistical analysis

Only subjects replaying all items were included. Descriptive information (mean, standard deviation, SD) were displayed for each item, and the overall score. The distribution of responses to individual items was also displayed, as is the percent of “always” responses to each item. Frequencies or percent for qualitative variables were also used. Student’s t test or analysis of variance (ANOVA) were used to compare continuous variables. Time to diagnosis was grouped into categories (0 to 3 years, 4 to 6, 7 to 15, and 16 or more). Chi-Square was used to compare qualitative and continuous variables. Given the overall descriptive nature of the results, no multiplicity adjustments were made. Lineal regression was calculated to establish the relation of age, number of drugs they are taking, number of times they have been admitted to the hospital in the last year, length of time in years that they have been diagnosed with the main illness (overall score on the PREM).

## Results

A total of 261 caregivers (in case of paediatric population) and patients with rare diseases (response rate 54.4%) answered, with a territorial distribution proportional to the population of the country as a whole. Of these, 232 (88.9%) adult patients and 29 (11.1%) caregivers of minor patients. The majority were males, 227 (87%), with an average age of 38 (SD 13.6) years. The mean time since confirmation of diagnosis was 7.8 (SD 8.0) years and, at the time of the study, they were taking an average of 4.3 (SD 3.3) medications daily (range 0–25). In general, the majority of patients were being treated regularly at the hospital (181, 69.3%) (Table 1). Patients and caregivers referred more than 50 different typologies of rare diseases.

**Table 1:** Description of the sample of subjects who answered to PREM

Sample age (mean, SD)	38 (13.6)
Age paediatric subsample (mean, SD)	10.1 (3.4)
Age adults subsample (mean, SD)	41.4 (10.1)
Sex	
Men (N, %)	227 (87.0)
Women (N, %)	34 (13.0)
Diagnosis	
The diagnosis is known (N, %)	239 (91.6)
Addison's disease (N, %)	24 (10)
Antiphospholipid syndrome (N, %)	9 (3.8)
Ehlers-Danlos syndrome (N, %)	8 (3.3)
Scleroderma (N, %)	4 (1.7)
The diagnosis is not yet known (N, %)	22 (8.4)
Time since the diagnosis is known (mean, SD)	7.8 (8.0)
Where they are usually receiving health care?	
Hospital (N, %)	181 (69.3)
Health center (N, %)	80 (30.7)
Number of drugs they are taking regularly, paediatric subsample (media, SD)	2.7 (2.4)
Number of times they have been admitted to hospital in the last year, paediatric subsample (media, SD)	0.9 (1.6)
Number of drugs they are taking regularly, adults subsample (media, SD)	4.5 (3.3)
Number of times they have been admitted to hospital in the last year, adults subsample (media, SD)	1.0 (2.3)

Overall, 39 (14.9%) participants confirmed the patient has received support from Social Services in the last six months, meanwhile 73 (28%) needed hospitalization, 153 (58.6%) had been seen in the emergency department and 21 (8%) were in the home care programme.

The score on the IEXPAC scale (11 elements) was 3.5 points (95%CI 3.2–3.8, SD 2.0). The systematic review of medication in each consultation was the element of care with the lowest range of improvement (Table 3). After hospital discharge, only 3 (4.1% of the total number of patients requiring hospitalization) said they had called or visited them at home to see how they were doing and what care they needed. Twenty out to 153 who had gone to the emergency department (13.1%) had been informed of which telephone number to call if they had an emergency again. Of the patients in home care, 6 (28.6%) considered that the professionals of the different health resources in which they received care were coordinated. Only 6 (16.8%) of the persons who had received care in the Social Services considered that there was adequate coordination of these professionals with those of the health services.

Neither the time of diagnosis, nor the number of hospital admissions, nor the number of medications they were taking influenced the patient's experience. As age increased, the patient's experience became more negative (Table 2).

**Table 2:** Relationship between age, number of medications they are taking, number of times they have been admitted to hospital in the last year, time in years they have been diagnosed with their main illness

	B	Standard error	*p* value	Lower endpoint	Upper endpoint
Age	− 0.03	0.01	0.001	− 0.05	− 0.01
Number of different medications you are taking	− 0.03	0.04	0.370	− 0.11	0.04
Number of times you have been admitted to hospital in the last year	0.05	0.05	0.387	− 0.06	0.15
Time in years you have been diagnosed with your main illness	0.02	0.01	0.249	− 0.01	0.05

Dependent variable: Experience Score IEXPAC RARE DISEASES

Caregivers of paediatric patients in all cases obtained higher scores on the PREM IEXPAC-rare-diseases scale, except for the coordination of social and health services (Table 3). In this case, all paediatric patients were treated in hospital paediatric services and when comparing their PREM IEXPAC rare-disease score with that of adult patients who usually treated in hospitals, the differences remain in favour of a better paediatric patient experience (paediatric age 5.2, SD 1.8; adult age 3.5, SD 1.9; T-Test = 4.3, *p* < 0.001). Patients routinely seen in hospitals instead of health centers scored higher on PREM IEXPAC-rare-diseases scale elements (Table 4). In general, females tended to reflect a better experience than males (Table 5).

**Table 3:** Score IEXPAC scale, paediatric subsample and adults subsample

	Total sample (N = 261)			Paediatric sample (N = 29)			Adult sample (N = 232)			*p* value
	Mean (CI95%)	SD	% = 5^+^	Mean (CI95%)	SD	% = 5^+^	Mean (CI95%)	SD	% = 5^+^	
1. They respect my lifestyle. The professionals who care for me listen to me and ask me about my needs, habits, and preferences to adapt my treatment and care plan	5.1 ± 0.3	2.8	11.5	6.6 ± 0.9	2.5	20.7	4.9 ± 0.4	2.8	10.3	0.002
2. They are coordinated to offer me good care. The professionals who care for me at the hospital, at the health center, and those who assisted me in my reference site talk to each other and coordinate to make sure that I receive diagnostic and coordinate to improve my wellbeing and quality of life	3.4 ± 0.4	3.2	6.9	5.2 ± 1.2	3.2	17.2	3.2 ± 0.4	3.2	5.6	0.002
3. They help me become informed via the Internet. The professionals who care for me inform me about reliable websited and Internet forums that I can consult to better understand my disease, its treatment, and the consequences that may have on my life	1.0 ± 0.3	2.3	2.7	2.0 ± 1.2	3.4	10.3	0.9 ± 0.3	2.1	1.7	0.014
4. I now know how to look after myself better. With the support of my professionals, I feel now that I have more confidence in my ability to take care of myself, manage my health problems and keep my autonomy	4.1 ± 0.3	2.9	6.9	5.3 ± 0.9	2.6	6.9	4.0 ± 0.4	2.9	6.9	0.016
5. They ask me about and help me follow my treatment plan. I review the adherence to my treatment and care plan with the professionals who care for me, and if I have questions, they answer them	5.3 ± 0.4	3.1	15.3	7.1 ± 0.9	2.4	24.1	5.1 ± 0.4	3.1	14.2	0.001
6. We agree on the most important objectives to a better manage of my disease. I have been able to agree with the professionals who care for me on which are the most important health and social problems and how to manage them properly to have the best quality of life	4.3 ± 0.4	3.1	10.0	6.9 ± 0.9	2.5	34.5	4.0 ± 0.4	3.0	6.9	0.001
7. I use the Internet and my mobile phone to consult my clinical record. I use the Internet and my mobile phone to consult my clinical record, test results, scheduled visits and to access other services on my health service’s website	2.4 ± 0.4	3.3	6.5	2.6 ± 1.3	3.5	10.3	2.3 ± 0.4	3.3	6.0	0.714
8. They ensure that I take my medication correctly. The professionals who care for me review with me all the medications I take, how I take them, how they make me feel, and I can ask them about the questions I have	4.9 ± 0.4	3.6	21.1	6.6 ± 1.4	3.7	44.8	4.7 ± 0.5	3.5	18.1	0.009
9. They are concerned about my wellbeing. The professionals who care for me are concerned with my quality of life and I feel they are committed to improving my wellbeing	5.1 ± 0.4	3.3	17.6	7.4 ± 1.0	2.6	41.4	4.8 ± 0.4	3.2	14.7	0.001
10. They inform me about health and social resources that can help me. The professionals who care for me inform me about the health and social resources available (in my neighbourhood, town or city) that I can use to improve my health problems	1.8 ± 0.3	2.6	2.3	3.5 ± 1.1	3.0	3.4	1.6 ± 0.3	2.5	2.2	0.001
11. They encourage me to talk with other patients. The professionals who care for me encourage me to participate in patients groups to share information and experiences on how to care for ourselves and improve our health	1.2 ± 0.3	2.3	1.1	3.3 ± 1.1	2.9	3.4	1.0 ± 0.3	2.0	0.9	0.001
Overall IEXPAC score	3.5 ± 0.6	2.2		5.1 ± 0.6	1.8		3.3 ± 0.2	1.9		0.001
12. My Health Service has identified a specialized center where I can be diagnosed and helped with my illness, to refer me there (N = 177)	3.3 ± 0.5	3.9	16.4	5.9 ± 1.7	4.3	44.0	2.9 ± 0.6	3.6	11.8	0.001
13. After being discharged from hospital, they have called or visited me at home to see how I was and what care I needed (N = 73)	0.9 ± 0.5	2.3	2.7	1.4 ± 1.8	2.4	0.0	0.8 ± 0.5	2.2	3.0	0.483
14. The professionals who attend me have given me a telephone number where I can contact if I have an emergency (N = 153)	2.0 ± 1.6	3.4	11.1	4.1 ± 2.9	4.9	36.4	1.8 ± 0.5	3.2	9.2	0.032
15. The professionals who care for me in my home try to solve my health problems in coordination with the professionals of the health center and the hospital (N = 21)	3.6 ± 1.0	3.8	14.3	6.3 ± 7.4	5.3	50.0	3.3 ± 1.7	3.7	10.5	0.312
16. The professionals who care for me in social services talk to and coordinate with the healthcare professionals to provide me with good care (N = 38)	2.6 ± 0.2	3.3	7.9	1.1 ± 1.5	2.0	0.0	2.9 ± 1.2	3.4	9.7	0.183

Comparison of scores

Score range 0 to 10

% = 5^+^ Percentage of subjects who answered with the always response option (score = 10)

**Table 4:** IEXPAC global score, subsample of the population attended at the health center versus the population attended at the hospital

	Health Center (N = 80)			Hospital (N = 181)			*p* value
	Mean (CI95%)	SD	% = 5^+^	Mean (CI95%)	SD	% = 5^+^	
1. They respect my lifestyle	4.4 ± 0.6	2.7	6.3	5.4 ± 0.4	2.8	13.8	0.005
2. They are coordinated to offer me good care	2.8 ± 0.7	3.0	6.3	3.7 ± 0.5	3.3	7.2	0.036
3. They help me become informed via the Internet	0.7 ± 0.4	1.9	1.3	1.1 ± 0.4	2.4	3.3	0.196
4. I now know how to look after myself better	3.7 ± 0.6	3.0	8.8	4.3 ± 0.4	2.8	6.1	0.102
5. They ask me about and help me follow my treatment plan	4.7 ± 0.7	3.2	13.8	5.6 ± 0.4	3.0	16.0	0.021
6. We agree on the most important objectives to a better manage of my disease	3.8 ± 0.7	3.1	10.0	4.6 ± 0.4	3.1	9.9	0.072
7. I use the Internet and my mobile phone to consult my clinical record	2.3 ± 0.7	3.2	3.8	2.4 ± 0.5	3.3	7.7	0.758
8. They ensure that I take my medication correctly	4.5 ± 0.7	3.2	13.8	5.1 ± 0.5	3.7	24.3	0.172
9. They are concerned about my wellbeing	4.2 ± 0.6	3.0	7.5	5.6 ± 0.5	3.3	22.1	0.001
10. They inform me about health and social resources that can help me	1.4 ± 0.5	2.3	1.3	2.0 ± 0.4	2.7	2.8	0.081
11. They encourage me to talk with other patients	1.1 ± 0.5	2.1	1.3	1.3 ± 0.3	2.3	11.1	0.413
Overall IEXPAC score	3.0 ± 0.4	1.9		3.7 ± 0.3	2.0		0.08
12. My Health Service has identified a specialized center where I can be diagnosed and helped with my illness, to refer me there (N = 177)	2.7 ± 0.9	3.5	10.9	3.6 ± 0.7	4.0	18.9	0.161
13. After being discharged from hospital, they have called or visited me at home to see how I was and what care I needed (N = 73)	1.2 ± 1.4	3.2	10.5	0.7 ± 0.5	1.9	0.0	0.464
14. The professionals who attend me have given me a telephone number where I can contact if I have an emergency (N = 153)	1.7 ± 0.9	3.2	9.8	2.1 ± 0.7	3.5	11.8	0.475
15. The professionals who care for me in my home try to solve my health problems in coordination with the professionals of the health center and the hospital (N = 21)	3.6 ± 2.1	3.6	9.1	3.5 ± 2.6	4.3	20.0	0.938
16. The professionals who care for me in social services talk to and coordinate with the healthcare professionals to provide me with good care (N = 38)	2.7 ± 1.9	3.5	7.7	2.5 ± 1.3	3.2	8.0	0.866

Comparison of scores

% = 5^+^ Percentage of subjects who answered with the always response option (score = 10)

**Table 5:** IEXPAC global score, subsample population of women versus men

	Men			Women			*p* value
	Mean (CI95%)	SD	% = 5^+^	Mean (CI95%)	SD	% = 5^+^	
1. They respect my lifestyle	5.0 ± 0.4	2.9	12.3	5.6 ± 0.7	2.1	5.9	0.286
2. They are coordinated to offer me good care	3.3 ± 0.4	3.3	7.5	4.5 ± 0.9	2.7	2.9	0.041
3. They help me become informed via the Internet	0.9 ± 0.3	2.2	2.6	1.7 ± 0.9	2.6	2.9	0.059
4. I now know how to look after myself better	4.0 ± 0.4	3.0	7.0	5.2 ± 0.8	2.3	5.9	0.018
5. They ask me about and help me follow my treatment plan	5.2 ± 0.4	3.2	15.4	6.4 ± 0.8	2.3	14.7	0.028
6. We agree on the most important objectives to a better manage of my disease	4.2 ± 0.4	3.1	10.6	5.1 ± 0.9	2.6	5.9	0.132
7. I use the Internet and my mobile phone to consult my clinical record	2.1 ± 0.4	3.2	5.7	3.9 ± 1.2	3.7	11.8	0.004
8. They ensure that I take my medication correctly	4.7 ± 0.5	3.5	18.5	6.4 ± 1.2	3.5	38.2	0.010
9. They are concerned about my wellbeing	5.0 ± 0.4	3.3	18.1	5.9 ± 0.9	2.7	14.7	0.151
10. They inform me about health and social resources that can help me	1.6 ± 0.3	2.6	2.6	3.1 ± 0.9	2.7	0.0	0.002
11. They encourage me to talk with other patients	1.1 ± 0.3	2.2	0.9	2.2 ± 0.9	2.7	2.9	0.007
Overall IEXPAC score	3.4 ± 0.2	2.2		4.5 ± 0.5	1.6		0.001
12. My Health Service has identified a specialized center where I can be diagnosed and helped with my illness, to refer me there (N = 177)	3.1 ± 0.6	3.8	14.2	4.5 ± 1.8	4.3	31.8	0.105
13. After being discharged from hospital, they have called or visited me at home to see how I was and what care I needed (N = 73)	0.6 ± 0.5	1.9	1.6	2.5 ± 2.2	3.5	10.0	0.012
14. The professionals who attend me have given me a telephone number where I can contact if I have an emergency (N = 153)	1.9 ± 0.5	3.2	9.5	2.8 ± 2.2	4.5	25.0	0.308
15. The professionals who care for me in my home try to solve my health problems in coordination with the professionals of the health center and the hospital (N = 21)	3.4 ± 2.0	4.2	18.8	4.0 ± 2.5	2.9	0.0	0.783
16. The professionals who care for me in social services talk to and coordinate with the healthcare professionals to provide me with good care (N = 38)	2.5 ± 1.2	3.3	10.3	2.8 ± 2.1	3.2	0.0	0.827

Comparison of scores

% = 5^+^ Percentage of subjects who answered with the always response option (score = 10)

When the results of this study are compared with those obtained by Orozco et al. and Guilabert et al., the results reflect that the experience of patients having a rare disease is highly worse (Table 6).

**Table 6:** PREM IEXPAC rare-disease scores compared with the results of IEXPAC global scores replied by patients having other chronic conditions (“Appendix 2”)

	Rare diseases		Chronic diseases (Orozco et al. [21])		Caregivers (Guilabert et al.[22])	
	Mean	% = 5^+^	Mean	% = 5^+^	Mean	% = 5^+^
1. They respect my lifestyle	5.1	2.8	8.3	56.5	7.8	45.5
2. They are coordinated to offer me good care	3.4	3.2	7.1	43.0	7.5	43.0
3. They help me become informed via the Internet	1.0	2.3	2.4	8.8	2.0	6.8
4. I now know how to look after myself better	4.1	2.9	8.1	47.6	7.3	38.7
5. They ask me about and help me follow my treatment plan	5.3	3.1	8.2	58.2	8.3	57.9
6. We agree on the most important objectives to a better manage of my disease	4.3	3.1	7.4	46.9	7.5	45.5
7. I use the Internet and my mobile phone to consult my clinical record	2.4	3.3	1.2	4.5	-	-
8. They ensure that I take my medication correctly	4.9	3.6	7.8	59.1	8.3	68.1
9. They are concerned about my wellbeing	5.1	3.3	8.5	65.6	8.3	60.0
10. They inform me about health and social resources that can help me	1.8	2.6	5.1	27.0	6.3	34.0
11. They encourage me to talk with other patients	1.2	2.3	2.6	9.0	3.5	18.7

% = 5^+^ Percentage of subjects who answered with the always response option (score = 10)

## Discussion

There are wide and important areas for improvement in the care of rare disease patients. This Spanish study confirms the results found in other countries [4, 6–10]. One of the common characteristics of the studies carried out to date, with Australian and North American populations with rare diseases, lies on the one hand in the delay in diagnosis and in the access to treatments [7, 9, 10]. Unmet health, social and emotional needs are a constant in studies on the experience of patients and caregivers with rare diseases [4, 6, 8]. A better understanding of these needs could improve the care paradigm. There is no positive experience with the organization of the assistance process in any of the aspects evaluated through the PREM IEXPAC-rare-diseases scale. In view of these results, and from the perspective of these patients, it cannot be considered that they receive integrated care and what is most striking, they do not have the perception that they receive enough support to manage their disease autonomously. These results highlight that the objective of empowering patients who suffer from a rare disease to face the social, psychological, occupational, etc. challenges of their illness and who go beyond their medical and health care needs are far from being met [12].

As far as we have been able to find out, this PREM instrument is the first in Spain focused on the experience of patients with rare diseases. The results are similar to the findings obtained in other countries and, compared to the results of similar studies conducted in Spain with another patient profile, they show that the experience of rare disease patients is even less positive.

Patients who are routinely seen in health centers, by primary care teams, described a more negative experience than those followed in hospitals. The greater proximity of primary care in this case does not seem to be a factor that contributes to a better experience. Conversely, patients seen in primary care who have responded do not feel that their lifestyle is sufficiently respected, they do not have the perception of having an individualized therapeutic plan in which they can get involved to achieve better results, they do not perceive that there is an adequate integration of the healthcare they receive and, more importantly, they do not have the feeling that professionals at this level of care are concerned about their well-being, if we compare with the results of patients usually seen in hospitals. The health services that have specialized units, where the diagnosis, follow-up and control of the disease is done, is where patients seem to show more confidence. These units have a multidisciplinary team that handles the case, which seems to influence patients and caregivers to refer a better experience.

Caregivers of paediatric patients who have been seen in hospitals report a better experience than adults, including comparison of those patients usually seen in hospitals. These results are relevant for several reasons. Firstly, because this comparison was not available. Secondly, because it is a more demanding population [9], families and caregivers of pediatric patients express greater concern for the patient's health than for their own. Third, because it allows further research to analyze in greater detail the process of care that paediatric services have put into practice to identify its key elements for a better experience in caregivers. Until now, there has been no comparison between the experience of patients with rare diseases and the experience of patients with chronic but high-prevalence diseases. In this case, the comparison is clearly negative for the group of patients with rare diseases, in line with other qualitative studies interviewing patients or their caregivers [4, 6]. The reasons that explain these results cannot be deduced from the data obtained but probably have to do with what has been suggested in other studies that analyse the interaction between patients and professionals in this particular case [23] and that highlight the fact that professionals do not always have adequate information and that they do not show styles of practice according to the communication needs of these patients.

These results seem to suggest that the proposal that this patient profile should have reference services, with staff sensitised to the psychological and social problems that accompany these disease processes[9], may contribute to a better experience in the course of the health care received. On the other hand, they also suggest that action plans, even if they arise from the health environment, should not forget the social care needs that some families may need. In this sense and according to these results, moving forward in the coordination between the two systems seems to be an objective for the action plans of the health organisations.

The following limitations should be considered when interpreting these results. First, that the selection of subjects was not random. It could have happened that people with the worst experience so far would have been encouraged to answer. Second, the subsample size of paediatric patients had a smaller number of informants than the adult subsample. This is also the case with the male sample that is over-represented. Third, the sample is only made up of patients and relatives enrolled in the FEDER who may have a different profile from the group of people living with rare diseases in Spain. Also, it must be considered the impact of variability in the disability associated to specific patients which is very different in a same disease. This study did not analized the impact of living in a urban area vs. rural one and some other more specific contextual information that could change deeply the perception of the being caring experience.

## Conclusions

This study is a first assessment of the experience of patients with rare diseases in Spain. Although its results cannot be generalized to other countries, there are aspects of qualitative and quantitative research that come to coincide and point to the need to design care processes for this patient profile considering the different information and communication needs of these patients / and their families) and that do not follow a pattern similar to that of patients (and their families) with frequent illnesses. Future research could analyze the elements of organization and professional care that promote a better patient experience.

## Data Availability

The datasets used and/or analyzed during the current study are available from the corresponding author on reasonable request.

## Appendix 1: Factor loadings of the PREM for rare diseases

**Table:**

Items included in the PREM-IEXPAC-rare-diseases instrument		
5. They ask me about and help me follow my treatment plan. I review the adherence to my treatment and care plan with the professionals who care for me, and if I have questions, they answer them	0.86	
9. They are concerned about my wellbeing. The professionals who care for me are concerned with my quality of life and I feel they are committed to improving my wellbeing	0.85	
6. We agree on the most important objectives to a better manage of my disease. I have been able to agree with the professionals who care for me on which are the most important health and social problems and how to manage them properly to have the best quality of life	0.81	
1. They respect my lifestyle. The professionals who care for me listen to me and ask me about my needs, habits, and preferences to adapt my treatment and care plan	0.80	
4. I now know how to look after myself better. With the support of my professionals, I feel now that I have more confidence in my ability to take care of myself, manage my health problems and keep my autonomy	0.72	
8. They ensure that I take my medication correctly. The professionals who care for me review with me all the medications I take, how I take them, how they make me feel, and I can ask them about the questions I have	0.72	
2. They are coordinated to offer me good care. The professionals who care for me at the hospital, at the health center, and those who assisted me in my reference site talk to each other and coordinate to make sure that I receive diagnostic and coordinate to improve my wellbeing and quality of life	0.69	
11. They encourage me to talk with other patients. The professionals who care for me encourage me to participate in patients groups to share information and experiences on how to care for ourselves and improve our health		0.76
3. They help me become informed via the Internet. The professionals who care for me inform me about reliable websited and Internet fórums that I can consult to better understand my disease, its treatment, and the consequences that may have on my life		0.65
10. They inform me about health and social resources that can help me. The professionals who care for me inform me about the health and social resources available (in my neighbourhood, town or city) that I can use to improve my health problems		0.63
7. I use the Internet and my mobile phone to consult my clinical record. I use the Internet and my mobile phone to consult my clinical record, test results, scheduled visits and to access other services on my health service’s website		0.55
Percentage of variance explained	41.9	17.4
Cronbach's Alpha	0.90	0.58

## Appendix 2: Statements IEXPAC rare diseases, chronic diseases, caregivers

**Table:**

Rare diseases	Chronic diseases	Caregivers
1. They respect my lifestyle. The professionals who care for me listen to me and ask me about my needs, habits, and preferences to adapt my treatment and care plan	1. They respect my lifestyle. The professionals who care for me listen to me and ask me about my needs, habits and preferences to adapt my treatment and care plan	1. They respect the lifestyle of the person I care for. The healthcare professionals who care for the person in my care ask me about their needs, habits and preferences to adapt their treatment and care plan
2. They are coordinated to offer me good care. The professionals who care for me at the hospital, at the health center, and those who assisted me in my reference site talk to each other and coordinate to make sure that I receive diagnostic and coordinate to improve my wellbeing and quality of life	2. They are coordinated to offer me good care. The professionals who care for me at the health centre and those who care for me at the hospital talk to each other and coordinate to improve my wellbeing and quality of life	2. They are coordinated to offer us good care. The healthcare professionals who care for the person in my care at the health centre and those who care for them at the hospital talk to each other and coordinate to improve their wellbeing and quality of life and those of the family
3. They help me become informed via the Internet. The professionals who care for me inform me about reliable websited and Internet forums that I can consult to better understand my disease, its treatment, and the consequences that may have on my life	3. They help me become informed via the Internet. The professionals who care for me inform me about reliable websites and Internet forums that I can consult to better understand my disease, its treatment and the consequences they may have on my life	3. They help me become informed via the Internet. The healthcare professionals who care for the person in my care inform me about websites and Internet forums that I can trust to better understand their disease, its treatment and the consequences they may have on their lives
4. I now know how to look after myself better. With the support of my professionals, I feel now that I have more confidence in my ability to take care of myself, manage my health problems and keep my autonomy	4. I now know how to look after myself better. With the support of my professionals, I feel now that I have more confidence in my ability to take care of myself, manage my health problems and keep my autonomy	4. I now know how to look after them better. With the support of the healthcare and social professionals caring for the person in my care, I feel I have more confidence in my ability to take care of them, manage their health problems and approach their situation better
5. They ask me about and help me follow my treatment plan. I review the adherence to my treatment and care plan with the professionals who care for me, and if I have questions, they answer them	5. They ask me about and help me follow my treatment plan. I review the adherence to my treatment and care plan with the professionals who care for me, and if I have questions, they answer them	5. They ask me about and help me follow the treatment plan of the person in my care. I review the adherence to their treatment and care plan with the healthcare professionals who care for the person in my care, and if I have questions, they answer them
6. We agree on the most important objectives to a better manage of my disease. I have been able to agree with the professionals who care for me on which are the most important health and social problems and how to manage them properly to have the best quality of life	6. We agree on objectives to lead a healthy life and to control my health problems better. I’ve been able to agree with the professionals who care for me on specific objectives regarding diet, physical exercise and medication to control my health problems better	6. We agree on the most important objectives of their care to control their health problems better. I’ve been able to discuss and agree with the healthcare professionals who care for the person in my care the most important health and social problems and how to manage them adequately to maintain their quality of life
7. I use the Internet and my mobile phone to consult my clinical record. I use the Internet and my mobile phone to consult my clinical record, test results, scheduled visits and to access other services on my health service’s website	7. I use the Internet and my mobile phone to consult my clinical record. I use the Internet and my mobile phone to consult my clinical record, test results, scheduled visits and to access other services on my health service’s website	–
8. They ensure that I take my medication correctly. The professionals who care for me review with me all the medications I take, how I take them, how they make me feel, and I can ask them about the questions I have	8. They ensure that I take my medication correctly. The professionals who care for me review with me all the medications I take, how I take them, how they make me feel, and I can ask them about the questions I have	7. They ensure that they take the medication correctly. The healthcare professionals caring for the person in my care review with me how to administer the medication and review with me if they are taking it correctly and how they are feeling
9. They are concerned about my wellbeing. The professionals who care for me are concerned with my quality of life and I feel they are committed to improving my wellbeing	9. They are concerned about my wellbeing. The professionals who care for me are concerned with my quality of life and I feel they are committed to improving my wellbeing	8. They are concerned about the wellbeing of the person in my care The healthcare and social care professionals who care for the person in my care are concerned about their quality of life and I feel they are committed to improving their wellbeing
10. They inform me about health and social resources that can help me. The professionals who care for me inform me about the health and social resources available (in my neighbourhood, town or city) that I can use to improve my health problems	10. They inform me about health and social resources that can help me The professionals who care for me inform me about the health and social resources available (in my neighbourhood, town or city) that I can use to improve my health problems and take better care of myself	11. They inform me about health and social resources that can help me. The healthcare and social care professionals who care for the person in my care inform me about the health and social resources available (in my neighbourhood, town or city) that I can use to improve the care I provide and to take better care of myself
11. They encourage me to talk with other patients. The professionals who care for me encourage me to participate in patients groups to share information and experiences on how to care for ourselves and improve our health	11. They encourage me to talk with other patients. The professionals who care for me encourage me to participate in patients groups to share information and experiences on how to care for ourselves and improve our health	12. They encourage me to talk to other caregiver. The healthcare and social care professionals who care for the person in my care encourage me to participate in caregiver groups to share information and experiences on how to care for ourselves and improve our competence as caregivers

## Abbreviations

PREM: Patient-Reported-Experience-Measure
FEDER: Federación Española de Enfermedades Raras
IEXPAC: Instrumento para evaluar la Experiencia del Paciente Crónico
